# Impact of Mir196a-2 Genotypes on Colorectal Cancer Risk in Taiwan

**DOI:** 10.3390/ijms241411613

**Published:** 2023-07-18

**Authors:** Te-Cheng Yueh, Yun-Chi Wang, Yu-Ting Chin, Yi-Chih Hung, Mei-Chin Mong, Ya-Chen Yang, Jen-Sheng Pei, Jian Gu, Chia-Wen Tsai, Da-Tian Bau, Wen-Shin Chang

**Affiliations:** 1 Division of Colon and Rectal Surgery, Department of Surgery, Taichung Armed Forces General Hospital, Taichung 41152, Taiwan; 2 National Defense Medical Center, Taipei 11490, Taiwan; 3 Graduate Institute of Biomedical Sciences, China Medical University, Taichung 404333, Taiwantim9711118888@gmail.com (Y.-T.C.);; 4 Terry Fox Cancer Research Laboratory, Department of Medical Research, China Medical University Hospital, Taichung 404327, Taiwan; 5 Department of Food Nutrition and Health Biotechnology, Asia University, Taichung 41354, Taiwan; 6 Department of Pediatrics, Taoyuan General Hospital, Ministry of Health and Welfare, Taoyuan 33004, Taiwan; 7 Department of Epidemiology, The University of Texas MD Anderson Cancer Center, Houston, TX 77030, USA; 8 Department of Bioinformatics and Medical Engineering, Asia University, Taichung 41354, Taiwan

**Keywords:** colorectal cancer, genotype, *mir146a*, *mir196a-2*, phenotype, polymorphism

## Abstract

We aimed to investigate the association between genotypes for *mir146a* and *mir196a-2* and the risk of developing colorectal cancer (CRC). We used polymerase chain reaction-restriction fragment length polymorphism (PCR-RFLP) to determine the *mir146a* rs2910164 and *mir196a-2* rs11614913 genotypes in 362 CRC patients and 362 controls. We also assessed the interactions between these genotypes and age, gender, smoking, alcohol consumption, and BMI status on CRC risk. Additionally, the serum expression level of mir196a-2 was quantified using quantitative reverse transcription-PCR. Our findings demonstrated that among the controls, the proportions of TT, CT, and CC genotypes of *mir196a-2* rs11614913 were 32.3%, 48.1%, and 19.6%, respectively. As for the cases, the proportions were 24.6%, 45.0%, and 30.4%, respectively. Logistic regression analysis revealed that the CC genotype carriers had a 2.04-fold increased risk (95% confidence interval [CI] = 1.36–3.06, *p* = 0.0008). Furthermore, carriers of the CT + CC genotypes also exhibited a significant association with CRC risk (odds ratio [OR] = 1.46, 95% CI = 1.06–2.03, *p* = 0.0261). Moreover, carriers of the CC genotype had significantly higher serum levels of mir196a-2 compared to those with the TT genotype (*p* < 0.0001), indicating a genotype-phenotype correlation. No association was found regarding *mir146a* rs2910164. In conclusion, *mir196a-2* rs2910164 genotypes, along with their associated expression, can serve as predictive markers for CRC risk.

## 1. Introduction

Colorectal cancer (CRC) is highly prevalent globally, ranking as the second most common cancer among women and the third most common among men [1,2,3]. The incidence and mortality rates of CRC vary significantly among countries, with differences of up to tenfold [2,3,4]. Several factors contribute to this variation, including meat consumption, cigarette smoking, and exposure to carcinogens, which account for approximately 85% of CRC cases [5,6].

In Taiwan, CRC is a significant health concern, with the highest incidence rate among all types of cancer and ranking third in terms of mortality, following lung cancer and hepatoma. The presence of a familial cancer history in approximately 15–20% of CRC cases [7,8], indicates the potential contribution of genetic factors to the development of CRC. In recent years, a plethora of genetic biomarkers associated with CRC have been identified [9,10,11,12,13,14], and there is still considerable interest in identifying additional genetic susceptibility factors and investigating the interactions between genetic factors and other risk factors. Gaining a better understanding of the genetic contributions to CRC can assist scientists in developing more targeted and precise approaches to cancer prevention and therapy.

MicroRNAs (miRNAs) are single-stranded, non-coding RNAs that function as negative regulators of the expression level of genes [15]. They play pivotal roles in diverse biological processes, encompassing embryonic development, cell proliferation, apoptosis, tissue remodeling, and notably, the process of carcinogenesis [16,17]. Genetic variations have been identified in both miRNA genes and their target genes, and these variations have been associated with a wide range of human diseases. Meanwhile, there is mounting evidence supporting the idea that dysregulated miRNAs play critical roles in tumorigenesis [18,19]. In CRC, several miRNAs have been identified to modulate cell proliferation, migration, apoptosis, and response to radiation in both CRC cells and patient samples. These miRNAs include miRNA 133A [20], miRNA 483-3p [21], miRNA 652 [22], and miRNA-627-5p [23]. Considering their dysregulation and significant correlation with numerous types of cancer, miRNAs emerge as promising targets for novel therapeutic strategies. Moreover, their secretion into extracellular fluids positions them as promising biomarkers for accessing tumor initiation, progression, metastasis, and tumor survival. However, the genetic contribution of miRNAs to the development of cancer has not yet been thoroughly investigated.

Single nucleotide polymorphisms (SNPs) are subtle genetic variations that commonly occur within miRNA genes. These genetic variations can potentially affect the expression and function of specific miRNAs, thereby contributing to tumorigenesis [24]. In the recent decade, accumulated studies have examined the relationship between numerous SNPs in miRNAs and the risk of CRC [25]. Among these SNPs, *mir146a* rs2910164 is the most extensively studied. The C allele of this SNP reduces the nuclear processing efficiency of pri-mir146a, resulting in a less stable structure and decreased mir146a expression [26]. In 2020, Santos and colleagues reported that mir146a expression within the tumor tissues was significantly higher among the CRC patients carrying the *mir146a* rs2910164 GG genotype compared to those carrying the GC or CC genotypes [27]. Regarding the association between this SNP and CRC risk, while the majority of the literature indicates no association [27,28,29,30,31,32,33,34], there are specific studies that have found associations within certain populations [35,36,37]. The CC genotype has been associated with increased CRC risks in populations from Greece [38], Korea [39], and China [35]. However, contrasting associations have been reported in Lithuania [40] and China [41,42]. Another frequently studied miRNA SNP is mir196a-2 rs11614913. Zhan and colleagues reported that this SNP influences RNA maturation and its interaction with other molecules [43]. Several studies have investigated its association with CRC risk, and the results have been inconsistent [28,29,30,31,33,37,38,40,42,43,44,45,46,47,48].

**Figure 1:**
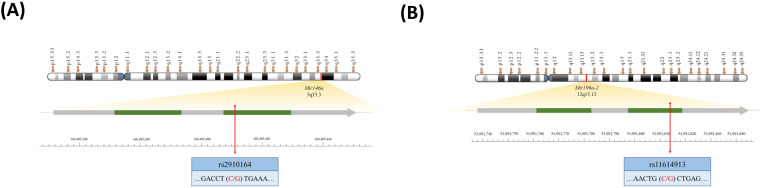
Physical maps of (**A**) *mir146a* rs2910164 (**A**) and (**B**) *mir196a-2* rs11614913 SNPs investigated in this study.

In this study, we aim to investigate the associations of the *mir146a* and *mir196a-2* SNPs with the risk of CRC among a Taiwanese population. The physical locations of these two SNPs are illustrated in Figure 1. Additionally, we aim to assess the potential interactions between genotypes and factors such as age, gender, smoking, and alcohol consumption in relation to CRC risk. Furthermore, we explored the correlation between genotypes and miRNA expression. Importantly, we conducted a thorough review of the existing literature and presented a concise discussion of its findings.

## 2. Results

### 2.1. Characteristics of Study Population

Table 1 presents the demographic and clinical characteristics of the cases and controls. The controls were 1:1 matched to the cases based on age and gender. There were no significant differences in the frequency of smoking (*p* = 0.543), alcohol consumption (*p* = 0.441), or BMI (*p* = 0.181) between the cases and controls.

### 2.2. Mir196a-2 Genotypes, but Not Mir146a Genotypes, Were Associated with CRC Risk

The *mir146a* rs2910164 and *mir196a-2* rs11614913 genotypic frequencies in the control groups fit well to the Hardy–Weinberg equation (*p* = 0.6631 and 0.1155, respectively). The distribution of genotypes does not show any significant differences among individuals with different characteristics (Supplemental Table S1). A significant association was observed between *mir196a-2* rs11614913 genotypes and the risk of CRC. Compared to the TT genotype, the OR for the heterozygous variant genotype CT carriers was 1.23 (95% CI = 0.87–1.75), whereas the homozygous variant genotype CC carriers were under a 2.04-fold increased CRC risk (OR = 2.04, 95% CI = 1.36–3.06) (*p* for trend = 0.0019). Individuals carrying the variant genotypes (CT + CC) had a 1.46-fold increased risk of CRC (OR = 1.46, 95% CI = 1.06–2.03) (Table 2). In contrast, there was no significant association between mir146a rs2910164 and CRC risk (Table 3).

### 2.3. Associations of Mir196a-2 and Mir146a Alleles with CRC Risk

Table 4 shows the allelic test of *mir146a* rs2910164 and *mir196a-2* rs11614913 in relation to CRC risk. The percentage of the G allele at *mir146a* rs2910164 in the control group was 45.5%, slightly higher than that (43.6%) observed in the CRC patient group. However, for *mir196a-2* rs11614913, the percentage of C allele was significantly higher in the CRC case (52.9%) than the control group (43.6%) (*p* = 0.0005). This corresponded to a 1.45-fold (95% CI = 1.18–1.78) increased risk of CRC for the C allele carriers compared to those of the T (OR = 1.45, 95% CI = 1.18–1.78) (Table 4).

### 2.4. Stratified Analyses of Mir196a-2 Genotypes by Age, Gender, Smoking, Alcohol Drinking Behaviors and BMI Status

We then conducted stratified analyses investigating the associations between *mir196a-2* rs11614913 with the risk of CRC based on differential age, gender, smoking, alcohol drinking behaviors, and BMI status (Table 5). Concisely, the associations between the *mir196a-2* rs11614913 SNP and CRC risk were statistically significant in all the strata, except for the smokers and drinkers. In the smoker and drinker subgroups, the risk for the CC genotype (OR = 2.11 and 2.36, 95% CI = 0.92–4.84 and 0.76–7.34, *p* = 0.1169 and 0.2239, respectively) did not reach a significant level. These findings cannot be settled down as a conclusion due to the small sample size tested.

### 2.5. Genotype-Phenotype Correlation of MiR196a-2 among Controls

**Figure 2:**
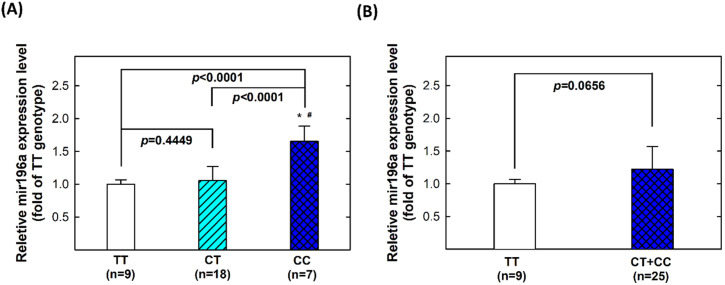
Correlation between *mir196a-2* genotypes and mir196a-2 expression in the serum of healthy subjects. (**A**) comparing three different genotypes; (**B**) comparing TT and CT + CC genotypes. * Statistically significant different from TT genotypes; # Statistically significant different from CT genotypes.

We further intended to investigate the functional levels of mir196a-2 expression in serum, and correlate the phenotypic patterns with their corresponding *mir196a-2* genotypes. For this purpose, we examined 34 serum samples from controls. Among these samples, 9 individuals had the TT genotype, 18 had the CT genotype, and 7 had the CC genotype at *mir196a-2* rs11614913. We observed a significant difference in the mean expression level of serum mir196a-2 among the three genotypes (*p* < 0.0001) in ANOVA analysis. In particular, individuals carrying the homozygous variant genotype CC had significantly higher levels of serum mir196a-2 compared with those carrying the TT genotype (*p* < 0.0001) (Figure 2A). When combining the CT and CC genotypes, the expression of mir196a-2 remained higher compared to the TT genotype, although the difference did not reach a significant level from the viewpoint of statistics (*p* = 0.0656) (Figure 2B).

## 3. Discussion

In this study, we provided evidence that the *mir196a-2* rs11614913 CC genotype and C allele may contribute to a higher risk of CRC (Table 2 and Table 4). The *mir146a* rs2910164 SNP was not associated with CRC risk in Taiwan (Table 3). Our findings are consistent with a previous study by Zhan, which reported an association between the *mir196a-2* rs11614913 C allele and increased CRC risk in China, [43]. However, two studies have reported conflicting results, suggesting that individuals carrying the *mir196a-2* rs11614913 TT genotypic pattern are at higher risk of developing CRC than those carrying the CC genotypic pattern in Iran and China [42,45]. The inconsistency between the findings of Zhan and Lv, despite studying similar populations, cannot be attributed to ethnic heterogeneity but rather may be influenced by sampling bias or other factors. It is worth noting that the CC genotype frequency in Lv’s study is unusually low. For Europeans, it appears that *mir196a-2* rs11614913 is not associated with CRC susceptibility [28,29,38,40]. For Japanese, the genotype of *mir196a-2* rs11614913 is not associated with CRC susceptibility either [31]. Thus far, the studies that have reported a positive association between *mir196a-2* rs11614913 and CRC risk have focused on Asian populations ([43] and the current study). The conflicting results observed in Iranian [45,48] and Chinese [42,43,47] populations may be resolved through further investigations involving larger sample sizes. It should be emphasized that the frequency of the T allele of mir196a-2 rs11614913 varies significantly across different ethnicities, ranging from 18.8% in Africans, 39.4% in Europeans, 41.1% in Mexicans, 30.7% in South Asians, and 54.8% in East Asians (Supplemental Table S2). The T allele frequency in our controls (56.4%) is similar to that in East Asians (54.8%). The T allele represents the major allele in East Asians but is the minor allele in all other ethnicities. Conducting additional studies on different populations could provide insights and help reconcile the discrepancies. All the literature investigating the associations between *mir196a-2* rs11614913 genotypes and CRC risk was summarized in Table 6, including the current study.

Quantification of mir196a-2 expression levels in serum can offer functional evidence that supports the role of mir196a-2 in the etiology of CRC. However, previous studies often lack the necessary data in this regard. Circulating miRNAs are believed to be encapsulated in exosomes, which protect them from degradation by RNase enzymes. Therefore, the expression of mir196a-2 could potentially serve as a measurable biomarker for CRC. In our study, we observed significantly higher expression levels of mir196a-2 carrying the homozygous variant genotype (CC) of *mir196a-2* rs11614913 compared to those carrying the wild-type TT genotype. This finding provides biological plausibility for the association of CC genotypes with increased CRC risk. The observed difference in RNA expression levels aligns with the genotypic data, supporting that the CC genotype has a significant effect on CRC risk. These findings are consistent with a previous report by Zhan, which reported higher expression levels of mir196a-2 in tumor tissue of patients carrying the CT and CC genotypes compared to those with the TT genotype [43].

The genotypes of *mir196a-2* rs11614913 may have a significant impact on various unresolved aspects of CRC. For example, several studies have examined the potential prognostic value of rs11614913 in Asian CRC patients. In 2018, Pao and colleagues showed that CRC patients carrying the rs11614913 CC genotype had a shorter overall survival time among 188 Taiwanese CRC cases [49]. Similarly, in 2011, Jang and colleagues reported that the heterozygous TC genotype, not the homozygous one, may serve as a risk factor for unfavorable overall survival in 446 Korean CRC patients [50]. Further investigations are necessary to validate these findings, as they were conducted exclusively in Asian populations. However, these findings hold potential clinical significance and emphasize the importance of exploring the prognostic value of mir196a-2 rs11614913 in other populations as well.

This study has several limitations that should be acknowledged. Firstly, the absence of CRC tissues prevented us from comparing mir196a-2 expression levels between CRC tissues and adjacent normal tissues. However, the measurements of serum mir196a-2 expression in controls provided an important genotype–phenotype (gene expression) correlation, supporting the observed association between genotypes and CRC risk. Secondly, the CRC patients included in our study were heterogeneous in terms of clinical features and treatments, and some patients were lost to follow-up, which hindered the analyses on the association between the mir196a-2 SNP and CRC prognosis. Thirdly, this investigation was conducted within a single medical center, China Medical University Hospital (CMUH). Although we have extinguished the possibility of population heterogeneity, further multi-center studies with larger sample sizes in the future are important to validate the current findings. Finally, given the small sample size, we only studied two SNPs. Future studies should include a more comprehensive, genome-wide analysis of SNPs in a significantly larger number of subjects.

In conclusion, the findings of the current study demonstrate that the C allele and CC genotype of *mir196a-2* rs11614913 are closely correlated to the increased CRC risk among the Taiwanese. Moreover, the CC genotype is correlated with significantly higher levels of serum mir196a-2 expression. Therefore, in conjunction with *mir196a-2* rs11614913 genotyping, the elevated serum levels of mir196a-2 may serve as a novel circulating marker for the early detection of CRC.

## 4. Materials and Methods

### 4.1. Study Population

The recruitment of CRC cases and healthy controls followed the same protocols as the routine sample collection work conducted in the Terry Fox Lab, as described in our previous papers [13,14]. Briefly, the CRC cases were recruited from CMUH from 2002 to 2008, and comprehensive pathological data, including staging, were accurately recorded. Controls were matched 1:1 to cases by age (with a difference ≤5 years) and gender. The age ranges for the patient and control groups were 42–89 and 44–90, respectively. The smokers include current and former smokers. Former smokers are those who have smoked at least 100 cigarettes in their lifetime but who have quit smoking for at least one year at the time of the interview. The drinkers include current and former drinkers. A current drinker is a person who consumed alcohol at least weekly in the year before the interview. Former drinkers are those who had quit drinking at least one year ago at the time of the interview. For each study participant, 10 mL of blood were collected and then delivered to the laboratory for DNA extraction and serum isolation within 24 h. The isolated DNA and serum were long-term stored at −80 °C and ready to use in this study. The study was approved by the Institutional Review Board of CMUH (coding number: DMR99-IRB-108). The staging status of the CRC patients was defined as Stage 1 to 4 for T1-2 N0 M0, T3-4 N0 M0, Tcarcinoma in situ-4 N1-2 M0, and Tcarcinoma in situ-4 N0-2 M1, respectively, according to the AJCC/UICC colorectal cancer staging classification standard.

### 4.2. Genotyping Methodology of Mir146a and Mir196a-2 Polymorphisms

Genomic DNA was extracted from the peripheral blood samples of each subject using a Qiagen kit (Qiagen, Chatsworth, CA, USA), according to the procedures described in our previous publication [51,52]. The genotyping of *mir146a* and *mir196a-2* polymorphic sites was conducted using the typical polymerase chain reaction restriction fragment length polymorphism (PCR-RFLP) method. For the *mir146a* rs2910164 polymorphism, the sequences of forward and reverse primers were 5′-CATGGGTTGTGTCAGTGTCAGAGCT-3′ and 5′-TGCCTTCTGTCTCCAGTCTTCCAA-3′, respectively. For the *mir196a-2* rs11614913 polymorphism, the sequences of forward and reverse primers are 5′-CCCCTTCCCTTCTCCTCCAGATA-3′ and 5′-CGAAAACCGACTGATGTAACTCCG-3′, respectively. PCR amplification was carried out using a PCR Thermocycler (Bio-RAD, Hercules, CA, USA) under the following conditions: initial denaturation at 94 °C for 5 min, followed by denaturation at 94 °C for 30 s, annealing at 64 °C for 40 s, and extension at 72 °C for 45 s. After 35 cycles of PCR, a final extension step was carried out at 72 °C for 10 min. The PCR amplicons for *mir146a* and *mir196a-2* were visualized after 3%-agarose gel 100-volt electrophoresis, and then digested with *Sac* I and *Msp* I restriction enzymes. The digestion products were subsequently verified through 4%-agarose gel 100-volt electrophoresis. The results of *mir146a* rs2910164 enzyme digestion presented three distinctive patterns: an unaltered single 147 bp fragment representing the GG genotype, fully digested fragments of 122 and 25 bp indicating the CC genotype, and fragments of 147, 122, and 25 bp representing the heterozygous GC genotype [53]. The results of *mir196a-2* rs11614913 enzyme digestion exhibited another three different patterns: an intact single 149 bp fragment representing the TT genotype, fully-digested fragments of 125 and 24 bp representing the homozygous variant CC genotype, and fragments of 149, 125, and 24 bp representing the heterozygous variant CT genotype [54].

### 4.3. Quantitative Reverse Transcription Polymerase Chain Reaction for Examining Mir196a-2 Transcriptional Expression

miRNA was extracted from serum using the miRNeasy Mini Isolation kit (Qiagen, Redwood, CA, USA) in accordance with the manufacturer’s instructions. Then, 1 μg of these miRNA samples were subsequently employed as templates for complementary DNA (cDNA) synthesis, utilizing the miScript II RT kit (Qiagen, Redwood, CA, USA). Then the reverse transcription (RT) reaction was conducted with the system set at: 42 °C for 15 min, 85 °C for 5 s, and then held at 4 °C. After the RT reaction was finished, the cDNA adducts were diluted at a 1:100 ratio, and 1 μL of the diluted cDNA adducts was subjected to the subsequent quantitative RT-PCR. For the quantification of mir196a-2 expression, the miScript SYBR Green PCR kit (Qiagen, Redwood City, CA, USA) was employed. All primers utilized were part of the SYBR green assays for mir196a-2 (Qiagen, Redwood City, CA, USA). The small nuclear RNA U6 was used as an internal control.

### 4.4. Statistical Analysis

The distributions of categorized age, gender, personal habits, different genotypes, and alleles among the subgroups were compared using Pearson’s chi-square test. The associations between different genotypes and CRC risk were assessed using individual odds ratios (ORs) with corresponding 95% confidence intervals (CIs). The serum mir196a-2 expression levels between different genotypes were compared with the unpaired Student’s *t*-test (for two groups, Figure 2B) and analysis of variance (ANOVA) (for three groups, Figure 2A). Statistical significance was defined as a *p*-Value less than 0.05.

## Figures and Tables

**Table 1:** Selected characteristics of the 362 CRC patients and 362 non-cancer controls.

Characteristic	Controls, *n* = 362		Cases, *n* = 362		*p*-Value ^a^
	*n*	%	*n*	%	
Age (years)					
≤60	95	26.2%	95	26.2%	1.0000
>60	267	73.8%	267	73.8%	
Gender					
Male	203	56.1%	203	56.1%	1.0000
Female	159	43.6%	159	43.9%	
Smoking					
Yes	84	23.2%	91	25.1%	0.5434
No	278	76.8%	271	74.9%	
Alcohol drinking					
Yes	51	14.1%	44	12.2%	0.4410
No	311	85.9%	318	87.8%	
BMI					
<24	175	48.3%	193	53.3%	0.1809
≥24	187	51.7%	169	46.7%	
Tumor size (cm)					
<5			195	53.9%	
≥5			167	46.1%	
Location					
Colon			257	71.0%	
Rectum			105	29.0%	
Lymph node involvement					
Negative			210	58.0%	
Positive			152	42.0%	
Stage					
Stage 1			94	26.0%	
Stage 2			72	19.9%	
Stage 3			134	37.0%	
Stage 4			62	17.1%	

SD, Standard deviation; BMI, body mass index; ^a^ based on Chi-square test with Yates’ correction.

**Table 2:** Associations between *mir196a-2* rs11614913 genotypes and CRC risk in Taiwan.

SNP	Genotype	Cases	Controls	*p*-Value	OR (95%CI)
rs11614913	TT	89 (24.6%)	117 (32.3%)		1.00 (Ref)
	CT	163 (45.0%)	174 (48.1%)	0.2792	1.23 (0.87–1.75)
	CC	110 (30.4%)	71 (19.6%)	0.0008 *	**2.04 (1.36–3.06)**
*P*_trend_				0.0019 *	
	CT + CC	273 (75.4%)	245 (67.7%)	0.0261 *	**1.46 (1.06–2.03)**
*P*_HWE_				0.6631	

OR: Odds ratio; CI: confidence interval; *p*-Values were calculated by Chi-square with Yates’ correction; HWE: Hardy–Weinberg Equilibrium; *P*_trend_, *p*-Value for trend analysis; *P*_HWE_, *p*-Value for Hardy–Weinberg equilibrium analysis *: *p* < 0.05; the significant parts are marked in bold.

**Table 3:** Associations between *mir146a* rs2910164 genotypes and CRC risk in Taiwan.

SNP	Genotype	Cases	Controls	*p*-Value	OR (95%CI)
rs2910164	CC	124 (34.3%)	119 (32.9%)		1.00 (Ref)
	CG	160 (44.2%)	164 (45.3%)	0.7618	0.94 (0.67–1.31)
	GG	78 (21.5%)	79 (21.8%)	0.8723	0.95 (0.63–1.42)
*P*_trend_				0.9237	
	CG + GG	238 (65.7%)	243 (67.1%)	0.7529	0.94 (0.69–1.28)
*P*_HWE_				0.1155	

OR: Odds ratio; CI: confidence interval; *p*-Values were calculated by Chi-square with Yates’ correction; HWE: Hardy–Weinberg Equilibrium; *P*_trend_, *p*-Value for trend analysis; *P*_HWE_, *p*-Value for Hardy–Weinberg equilibrium analysis.

**Table 4:** Associations of *mir146a* rs2910164 and *mir196a-2* rs11614913 alleles with CRC risk.

Allele	Cases	Controls	*p*-Value	OR (95%CI)
*mir146a* rs2910164				
C	408 (56.4%)	402 (55.5%)		1.00 (Ref)
G	316 (43.6%)	322 (45.5%)	0.7913	0.97 (0.79–1.19)
*mir196a-2* rs11614913				
T	341 (47.1%)	408 (56.4%)		1.00 (Ref)
C	383 (52.9%)	316 (43.6%)	0.0005 *	**1.45 (1.18–1.78)**

*p*-Value was calculated by Chi-square with Yates’ correction; *: *p* < 0.05; the significant parts are marked in bold.

**Table 5:** Associations between *mir196a-2* rs11614913 genotypes and CRC risk in stratified analyses.

Characteristics	Controls	Cases	OR (95% CI) ^a^	aOR (95% CI) ^b^	*p*-Value ^c^
Age					
≤60 years old					
TT	32	23	1.00 (ref)	1.00 (ref)	
CT	48	43	1.25 (0.63–2.45)	1.21 (0.67–2.39)	0.6400
CC	15	29	**2.69 (1.18–6.12)**	**2.77 (1.23–4.98)**	0.0291 *
>60 years old					
TT	85	66	1.00 (ref)	1.00 (ref)	
CT	126	120	1.23 (0.82–1.84)	1.33 (0.79–2.11)	0.3791
CC	56	81	**1.86 (1.17–2.98)**	**2.04 (1.24–3.58**)	0.0126 *
Gender					
Males					
TT	69	52	1.00 (ref)	1.00 (ref)	
CT	94	89	1.26 (0.79–1.99)	1.19 (0.81–1.86)	0.3948
CC	40	62	**2.06 (1.20–3.52)**	**2.28 (1.19–3.23)**	0.0119 *
Females					
TT	48	37	1.00 (ref)	1.00 (ref)	
CT	80	74	1.20 (0.70–2.04)	1.19 (0.74–2.17)	0.5922
CC	31	48	**2.01 (1.08–3.74)**	**2.04 (1.12–3.88)**	0.0404 *
Smoking behaviors					
Non-smokers					
TT	91	68	1.00 (ref)	1.00 (ref)	
CT	133	122	1.23 (0.82–1.83)	1.31 (0.92–2.53)	0.3646
CC	54	81	**2.01 (1.26–3.20)**	**2.27 (1.23–2.97)**	0.0047 *
Smokers					
TT	26	21	1.00 (ref)	1.00 (ref)	
CT	41	41	1.24 (0.60–2.54)	1.27 (0.58–2.49)	0.6900
CC	17	29	2.11 (0.92–4.84)	2.16 (0.97–4.38)	0.1169
Alcohol drinking behaviors					
Non-drinkers					
TT	99	78	1.00 (ref)	1.00 (ref)	
CT	150	143	1.21 (0.83–1.76)	1.28 (0.85–2.04)	0.3672
CC	62	97	**1.99 (1.28–3.07)**	**2.04 (1.17–2.89)**	0.0028 *
Drinkers					
TT	18	11	1.00 (ref)	1.00 (ref)	
CT	24	20	1.36 (0.52–3.55)	1.29 (0.48–3.42)	0.6933
CC	9	13	2.36 (0.76–7.34)	2.24 (0.74–6.93)	0.2239
BMI					
<24					
TT	57	49	1.00 (ref)	1.00 (ref)	
CT	81	86	1.24 (0.76–2.01)	1.22 (0.73–1.98)	0.4686
CC	37	58	**1.82 (1.04–3.20)**	**1.84 (1.09–3.35)**	0.0498 *
≥24					
TT	60	40	1.00 (ref)	1.00 (ref)	
CT	93	77	1.24 (0.75–2.05)	1.21 (0.72–2.34)	0.4712
CC	34	52	**2.29 (1.27–4.13)**	**2.38 (1.34–3.97)**	0.0084 *

^a^, by univariate logistic regression analysis; ^b,c^ by multivariate logistic regression analysis after the adjustments of confounding factors; CI, confidence interval; aOR, adjusted odds ratio; *: *p* < 0.05; the significant parts are marked in bold.

**Table 6:** Literature reports of the associations between *mir196a-2* rs11614913 genotypes and CRC risk.

First Author	Year	Ethnicity	TT, CT, CC Genotype # of the Controls	TT, CT, CC Genotype # of the Cases	OR (95%CI) ^a^	*p*-Value ^b^	Ref #
Chen	2011	Chinese	107:206:94	35:64:27	0.94 (0.71–1.25)	0.715	[47]
Zhan	2011	Chinese	163:267:113	56:128:68	1.32 (1.07–1.64)	0.011	[43]
Min	2012	Korean	148:254:100	125:201:120	1.19 (0.99–1.42)	0.073	[37]
Hezova	2012	European	22:103:87	26:89:82	0.95 (0.71–1.27)	0.811	[28]
Vinci	2013	European	11:84:83	12:86:62	0.81 (0.59–1.12)	0.231	[29]
Lv	2013	Chinese	91:331:109	114:223:10	0.50 (0.41–0.61)	<0.001	[42]
Kupcinskas	2014	European	54:174:199	27:87:79	0.86 (0.67–1.10)	0.254	[40]
Parlayan	2014	Japanese	390:679:282	34:59:23	0.97 (0.74–1.27)	0.881	[31]
Dikaiakos	2015	European	117:149:33	69:69:19	0.92 (0.69–1.23)	0.624	[38]
Chayeb	2018	Tunisian	29:85:47	31:82:39	0.89 (0.65–1.22)	0.508	[30]
Haerian	2018	Iranian	187:551:505	262:196:449	0.90 (0.80–1.02)	0.105	[45]
Soltanian	2021	Iranian	56:122:108	29:91:74	1.11 (0.85–1.45)	0.477	[48]
Yueh	2023	Taiwanese	117:174:71	89:163:110	1.45 (1.18–1.78)	0.001	current

OR: Odds ratio; CI: confidence interval; Ref #: Reference number; ^a^ C allele versus T allele at *mir196a-2* rs11614913; ^b^ *p*-Values were calculated by Chi-square with Yates’ correction.

## Data Availability

The genotyping results and clinical data supporting the findings of this study are available from the corresponding authors upon reasonable requests via email at artbau2@gmail.com.

## Supplementary Materials

The supporting information can be downloaded at: https://www.mdpi.com/article/10.3390/ijms241411613/s1.

## References

[1] Siegel R.L., Wagle N.S., Cercek A., Smith R.A., Jemal A. (2023). Colorectal cancer statistics, 2023. CA Cancer J. Clin..

[2] Sung H., Ferlay J., Siegel R.L., Laversanne M., Soerjomataram I., Jemal A., Bray F. (2021). Global Cancer Statistics 2020: GLOBOCAN Estimates of Incidence and Mortality Worldwide for 36 Cancers in 185 Countries. CA Cancer J. Clin..

[3] You L., Lv Z., Li C., Ye W., Zhou Y., Jin J., Han Q. (2021). Worldwide cancer statistics of adolescents and young adults in 2019: A systematic analysis of the Global Burden of Disease Study 2019. ESMO Open.

[4] Dong W., Kim U., Rose J., Hoehn R.S., Kucmanic M., Eom K., Li S., Berger N.A., Koroukian S.M. (2023). Geographic Variation and Risk Factor Association of Early Versus Late Onset Colorectal Cancer. Cancers.

[5] Agache A., Mustatea P., Mihalache O., Bobirca F.T., Georgescu D.E., Jauca C.M., Birligea A., Doran H., Patrascu T. (2018). Diabetes Mellitus as a Risk-factor for Colorectal Cancer Literature Review—Current Situation and Future Perspectives. Chirurgia.

[6] Li N., Lu B., Luo C., Cai J., Lu M., Zhang Y., Chen H., Dai M. (2021). Incidence, mortality, survival, risk factor and screening of colorectal cancer: A comparison among China, Europe, and northern America. Cancer Lett..

[7] Kao P.S., Lin J.K., Wang H.S., Yang S.H., Jiang J.K., Chen W.S., Lin T.C., Li A.F., Liang W.Y., Chang S.C. (2009). The impact of family history on the outcome of patients with colorectal cancer in a veterans’ hospital. Int. J. Colorectal Dis..

[8] Yeh Y.L., Li M., Kwok O.M., Ma P., Chen L.S. (2022). Chinese Americans’ Family History of Colorectal Cancer Communication with Primary Care Physicians. Health Educ. Behav..

[9] Hung Y.C., Chang W.S., Chou A.K., Pei J.S., Yang M.D., Yang H.R., Yang T.M., Wang Y.C., Hsiau Y.C., Chen C.P. (2020). Association of Adiponectin Genotypes with Colorectal Cancer Susceptibility in Taiwan. Anticancer Res..

[10] Wu M.H., Chen C.H., Chen C.P., Huang T.L., Yueh T.C., Wang Z.H., Tsai C.W., Pei J.S., Mong M.C., Yang Y.C. (2022). Contribution of 5-Methyltetrahydrofolate-Homocysteine Methyltransferase Reductase Genotypes to Colorectal Cancer in Taiwan. Anticancer Res..

[11] Wu M.H., Hung Y.W., Gong C.L., Chao C.C., Yueh T.C., Wang S.C., Lai Y.L., Hsu S.W., Fu C.K., Wang Y.C. (2019). Contribution of Caspase-8 Genotypes to Colorectal Cancer Risk in Taiwan. Anticancer Res..

[12] Wu M.H., Tzeng H.E., Wu C.N., Yueh T.C., Peng Y.C., Tsai C.H., Wang Y.C., Ke T.W., Pei J.S., Chang W.S. (2019). Association of Matrix Metalloproteinase-9 rs3918242 Promoter Genotypes with Colorectal Cancer Risk. Anticancer Res..

[13] Wu M.H., Yueh T.C., Chang W.S., Tsai C.W., Fu C.K., Yang M.D., Yu C.C., Bau D.T. (2021). Contribution of Matrix Metalloproteinase-1 Genotypes to Colorectal Cancer in Taiwan. Cancer Genom. Proteom..

[14] Yueh T.C., Hung Y.C., Lee H.T., Yang M.D., Wang Z.H., Yang Y.C., Ke T.W., Pei J.S., Tsai C.W., Bau D.T. (2022). Role of Matrix Metallopeptidase-2 Genotypes in Taiwanese Patients with Colorectal Cancer. Anticancer Res..

[15] Devara D., Choudhary Y., Kumar S. (2023). Role of MicroRNA-502-3p in Human Diseases. Pharmaceuticals.

[16] Kim W.R., Park E.G., Lee D.H., Lee Y.J., Bae W.H., Kim H.S. (2023). The Tumorigenic Role of Circular RNA-MicroRNA Axis in Cancer. Int. J. Mol. Sci..

[17] Tajik F., Alian F., Yousefi M., Azadfallah A., Hoseini A., Mohammadi F., Karimi-Dehkordi M., Alizadeh-Fanalou S. (2023). MicroRNA-372 acts as a double-edged sword in human cancers. Heliyon.

[18] Kontham S.S., Walter C.E.J., Shankaran Z.S., Ramanathan A., Karuppasamy N., Johnson T. (2022). A microRNA binding site polymorphism in the 3′ UTR region of VEGF-A gene modifies colorectal cancer risk based on ethnicity: A meta-analysis. J. Egypt Natl. Canc. Inst..

[19] Wang Z., Zhu X., Zhai H., Wang Y., Hao G. (2022). Integrated analysis of mRNA-single nucleotide polymorphism-microRNA interaction network to identify biomarkers associated with prostate cancer. Front. Genet..

[20] Sharma G., Mo J.S., Lamichhane S., Chae S.C. (2023). MicroRNA 133A Regulates Cell Proliferation, Cell Migration, and Apoptosis in Colorectal Cancer by Suppressing CDH3 Expression. J. Cancer.

[21] Candiello E., Reato G., Verginelli F., Gambardella G., Ambrosio D.A., Calandra N., Orzan F., Iuliano A., Albano R., Sassi F. (2023). MicroRNA 483-3p overexpression unleashes invasive growth of metastatic colorectal cancer via NDRG1 downregulation and ensuing activation of the ERBB3/AKT axis. Mol. Oncol..

[22] Pathak S., Meng W.J., Sriramulu S., Jothimani G., Jangamreddy J.R., Banerjee A., Ganesan A.T., Adell G., Zhang X., Sun Zhang A. (2023). Association of microRNA-652 expression with radiation response of colorectal cancer: A study from rectal cancer patients in a Swedish trial of preoperative radiotherapy. Curr. Gene Ther..

[23] Zhao D.Y., Yin T.F., Sun X.Z., Zhou Y.C., Wang Q.Q., Zhou G.Y., Yao S.K. (2023). microRNA-627-5p inhibits colorectal cancer cell proliferation, migration and invasion by targeting Wnt2. World J. Gastrointest. Oncol..

[24] Wang D., Wang Y., Lin Z., Cai L. (2020). Association between miRNA-146a polymorphism and lung cancer susceptibility: A meta-analysis involving 6506 cases and 6576 controls. Gene.

[25] Li X.Y., Huang Z.J., Li J.P., Gao X.R. (2023). rs3735664 Polymorphism Affecting ELFN1-AS1 Adsorption on miR-1231 is Associated with Colorectal Cancer Susceptibility and Tumor Stage. Biomed. Environ. Sci..

[26] Jazdzewski K., Murray E.L., Franssila K., Jarzab B., Schoenberg D.R., de la Chapelle A. (2008). Common SNP in pre-miR-146a decreases mature miR expression and predisposes to papillary thyroid carcinoma. Proc. Natl. Acad. Sci. USA.

[27] Santos J.S.D., Zunta G.L., Negrini A.B., Ribeiro M.S.G., Martinez C.A.R., Ribeiro M.L., Lourenco G.J., Ortega M.M. (2020). The association of a single-nucleotide variant in the microRNA-146a with advanced colorectal cancer prognosis. Tumour Biol..

[28] Hezova R., Kovarikova A., Bienertova-Vasku J., Sachlova M., Redova M., Vasku A., Svoboda M., Radova L., Kiss I., Vyzula R. (2012). Evaluation of SNPs in miR-196-a2, miR-27a and miR-146a as risk factors of colorectal cancer. World J. Gastroenterol..

[29] Vinci S., Gelmini S., Mancini I., Malentacchi F., Pazzagli M., Beltrami C., Pinzani P., Orlando C. (2013). Genetic and epigenetic factors in regulation of microRNA in colorectal cancers. Methods.

[30] Chayeb V., Mahjoub S., Zitouni H., Jrah-Harzallah H., Zouari K., Letaief R., Mahjoub T. (2018). Contribution of microRNA-149, microRNA-146a, and microRNA-196a2 SNPs in colorectal cancer risk and clinicopathological features in Tunisia. Gene.

[31] Parlayan C., Ikeda S., Sato N., Sawabe M., Muramatsu M., Arai T. (2014). Association analysis of single nucleotide polymorphisms in miR-146a and miR-196a2 on the prevalence of cancer in elderly Japanese: A case-control study. Asian Pac. J. Cancer Prev..

[32] Ying H.Q., Peng H.X., He B.S., Pan Y.Q., Wang F., Sun H.L., Liu X., Chen J., Lin K., Wang S.K. (2016). MiR-608, pre-miR-124-1 and pre-miR26a-1 polymorphisms modify susceptibility and recurrence-free survival in surgically resected CRC individuals. Oncotarget.

[33] Lindor N.M., Larson M.C., DeRycke M.S., McDonnell S.K., Baheti S., Fogarty Z.C., Win A.K., Potter J.D., Buchanan D.D., Clendenning M. (2017). Germline miRNA DNA variants and the risk of colorectal cancer by subtype. Genes Chromosomes Cancer.

[34] Jiang J., Zhang S., Tang W., Qiu Z. (2021). Lack of association between miR-146a rs2910164 C/G locus and colorectal cancer: From a case-control study to a meta-analysis. Biosci. Rep..

[35] Mao Y., Li Y., Jing F., Cai S., Zhang Z., Li Q., Ma X., Wang J., Jin M., Chen K. (2014). Association of a genetic variant in microRNA-146a with risk of colorectal cancer: A population-based case-control study. Tumour Biol..

[36] Gao X., Zhu Z., Zhang S. (2018). miR-146a rs2910164 polymorphism and the risk of colorectal cancer in Chinese population. J. Cancer Res. Ther..

[37] Min K.T., Kim J.W., Jeon Y.J., Jang M.J., Chong S.Y., Oh D., Kim N.K. (2012). Association of the miR-146aC>G, 149C>T, 196a2C>T, and 499A>G polymorphisms with colorectal cancer in the Korean population. Mol. Carcinog..

[38] Dikaiakos P., Gazouli M., Rizos S., Zografos G., Theodoropoulos G.E. (2015). Evaluation of genetic variants in miRNAs in patients with colorectal cancer. Cancer Biomark..

[39] Chae Y.S., Kim J.G., Lee S.J., Kang B.W., Lee Y.J., Park J.Y., Jeon H.S., Park J.S., Choi G.S. (2013). A miR-146a polymorphism (rs2910164) predicts risk of and survival from colorectal cancer. Anticancer Res..

[40] Kupcinskas J., Bruzaite I., Juzenas S., Gyvyte U., Jonaitis L., Kiudelis G., Skieceviciene J., Leja M., Pauzas H., Tamelis A. (2014). Lack of association between miR-27a, miR-146a, miR-196a-2, miR-492 and miR-608 gene polymorphisms and colorectal cancer. Sci. Rep..

[41] Ma L., Zhu L., Gu D., Chu H., Tong N., Chen J., Zhang Z., Wang M. (2013). A genetic variant in miR-146a modifies colorectal cancer susceptibility in a Chinese population. Arch. Toxicol..

[42] Lv M., Dong W., Li L., Zhang L., Su X., Wang L., Gao L., Zhang L. (2013). Association between genetic variants in pre-miRNA and colorectal cancer risk in a Chinese population. J. Cancer Res. Clin. Oncol..

[43] Zhan J.F., Chen L.H., Chen Z.X., Yuan Y.W., Xie G.Z., Sun A.M., Liu Y. (2011). A functional variant in microRNA-196a2 is associated with susceptibility of colorectal cancer in a Chinese population. Arch. Med. Res..

[44] Zhu L., Chu H., Gu D., Ma L., Shi D., Zhong D., Tong N., Zhang Z., Wang M. (2012). A functional polymorphism in miRNA-196a2 is associated with colorectal cancer risk in a Chinese population. DNA Cell Biol..

[45] Haerian M.S., Haerian B.S., Molanaei S., Kosari F., Sabeti S., Bidari-Zerehpoosh F., Abdolali E. (2018). MIR196A2 rs11614913 contributes to susceptibility to colorectal cancer in Iranian population: A multi-center case-control study and meta-analysis. Gene.

[46] Toraih E.A., Fawzy M.S., Mohammed E.A., Hussein M.H., El-Labban M.M. (2016). MicroRNA-196a2 Biomarker and Targetome Network Analysis in Solid Tumors. Mol. Diagn. Ther..

[47] Chen H., Sun L.Y., Chen L.L., Zheng H.Q., Zhang Q.F. (2012). A variant in microRNA-196a2 is not associated with susceptibility to and progression of colorectal cancer in Chinese. Intern. Med. J..

[48] Soltanian A.R., Hosseini B., Mahjub H., Bahreini F., Nazemalhosseini Mojarad E., Ghaffari M.E. (2021). Association between rs11614913 Polymorphism of The MiR-196-a2 Gene and Colorectal Cancer in The Presence of Departure from Hardy-Weinberg Equilibrium. Cell J..

[49] Pao J.B., Lu T.L., Ting W.C., Chen L.M., Bao B.Y. (2018). Association of Genetic Variants of Small Non-Coding RNAs with Survival in Colorectal Cancer. Int. J. Med. Sci..

[50] Jang M.J., Kim J.W., Min K.T., Jeon Y.J., Oh D., Kim N.K. (2011). Prognostic significance of microRNA gene polymorphisms in patients with surgically resected colorectal cancer. Exp. Ther. Med..

[51] Yang M.D., Lin K.C., Lu M.C., Jeng L.B., Hsiao C.L., Yueh T.C., Fu C.K., Li H.T., Yen S.T., Lin C.W. (2017). Contribution of matrix metalloproteinases-1 genotypes to gastric cancer susceptibility in Taiwan. Biomedicine.

[52] Tsai C.W., Chang W.S., Xu Y., Huang M., Bau D.T., Gu J. (2021). Associations of genetically predicted circulating insulin-like growth factor-1 and insulin-like growth factor binding protein-3 with bladder cancer risk. Mol. Carcinog..

[53] Pei J.S., Chang W.S., Hsu P.C., Chen C.C., Chin Y.T., Huang T.L., Hsu Y.N., Kuo C.C., Wang Y.C., Tsai C.W. (2020). Significant Association Between the MiR146a Genotypes and Susceptibility to Childhood Acute Lymphoblastic Leukemia in Taiwan. Cancer Genom. Proteom..

[54] Chen C.C., Hsu P.C., Shih L.C., Hsu Y.N., Kuo C.C., Chao C.Y., Chang W.S., Tsai C.W., Bau D.T., Pei J.S. (2020). MiR-196a-2 Genotypes Determine the Susceptibility and Early Onset of Childhood Acute Lymphoblastic Leukemia. Anticancer Res..

