# Supplementary material for: Impact of Mir196a-2 Genotypes on Colorectal Cancer Risk in Taiwan
Source: Int J Mol Sci. 2023 Jul 18;24(14):11613. doi: 10.3390/ijms241411613 (PMC10380589; doi:10.3390/ijms241411613)
Supplement: Supplementary file 1 [file ijms-24-11613-s001.zip › ijms-2432527-SI.pdf]

Supplemental Table S1. Correlation between *mir196a-2* rs11614913 genotype and epidemiological characteristics

Index	Subgroup	Controls, n	*mir196a-2* rs11614913 genotype, n (%)			*p*-Value[a]
			TT	CT	CC	
Age	≤60 Years	95	32 (33.7)	48 (50.5)	15 (15.8)	0.5498
	>60 Years	267	85 (31.8)	126 (47.2)	56 (21.0)	
Gender	Male	203	69 (34.0)	94 (46.3)	40 (19.7)	0.7051
	Female	159	48 (30.2)	80 (50.3)	31 (19.5)	
Cigarette smoker	Ever	278	91 (32.8)	133 (47.8)	54 (19.4)	0.9523
	Never	84	26 (31.0)	41 (48.8)	17 (20.2)	
Alcohol drinker	Ever	311	99 (31.8)	150 (48.2)	62 (20.0)	0.8642
	Never	51	18 (35.3)	24 (47.1)	9 (17.6)	
BMI	<24	175	57 (32.6)	81 (46.3)	37 (21.1)	0.7283
	≥24	187	60 (32.1)	93 (49.7)	34 (18.2)	

[a]Based on Fisher’s exact test.

Supplemental Table S2. Variant (T) allele frequencies of mir196a-2 rs11614913 in different populations

SNP	Population	Sample size (Healthy controls)	Variant (T) allele frequency
rs11614913			
	European	190002	0.394
	African	41930	0.188
	Mexican	10808	0.411
	European American	7164	0.407
	Central American	2450	0.354
	South American	1982	0.363
	South Asian	4984	0.307
	East Asian	3118	0.548
	Taiwanese (current study)	362	0.564

Data were extracted from https://www.ncbi.nlm.nih.gov/snp/
